# Accuracy of WHO Verbal Autopsy Tool in Determining Major Causes of Neonatal Deaths in India

**DOI:** 10.1371/journal.pone.0054865

**Published:** 2013-01-25

**Authors:** Arun K. Aggarwal, Praveen Kumar, Sadbhawna Pandit, Rajesh Kumar

**Affiliations:** 1 School of Public Health, PGIMER, Chandigarh, India; 2 Neonatal Unit, Department of Pediatrics, Advanced Pediatrics Center, PGIMER, Chandigarh, India; 3 Department of Pediatrics, Govt Multispecialty Hospital, Sector 16, Chandigarh, India; Tulane University School of Public Health and Tropical Medicine, United States of America

## Abstract

**Objectives:**

This study was conducted to evaluate the performance of World Health Organisation (WHO) verbal autopsy tool in determining major causes of neonatal deaths.

**Methods:**

From a tertiary care hospital and a government multispecialty hospital, the attending paediatricians ascertained a clinical cause of death for 371 neonatal deaths. Trained field workers conducted verbal autopsy (VA) interviews. Two independent paediatricians, who had no access to the clinical information, assigned cause of death as per verbal autopsy. Analysis was based on 313 cases in which both clinical diagnosis and VA diagnosis was obtained.

**Findings:**

As per the clinical diagnosis, four most common causes of neonatal deaths were sepsis (29.1%), preterm birth (27.8%), birth asphyxia (27.2%), and congenital anomalies (11.5%). Cause specific mortality fractions by VA diagnosis were statistically similar to those obtained by clinical diagnosis except for birth asphyxia (16.3%). Diagnostic accuracy of verbal autopsy diagnosis against clinical diagnosis ranged from 78% to 92% in ascertaining different underlying causes of death. Area under the Receiver-Operator Characteristics curve (95% confidence interval) was 0.75 (0.69–0.80) for sepsis, 0.74 (0.68–0.80) for preterm birth, 0.73 (0.65–0.82) for congenital anomaly and 0.70 (0.64–0.75) for birth asphyxia. Kappa for all four causes was moderate (0.46–0.55).

**Interpretation:**

The WHO verbal autopsy tools can provide reasonably good estimates of predominant causes of neonatal deaths in countries where neonatal mortality is high. Caution is required to interpret cause specific mortality fraction (CSMF) for birth asphyxia by VA because it is likely to be an underestimate.

## Introduction

Globally, about 4 million neonatal deaths occur every year [1]. Direct causes of neonatal deaths are estimated to be preterm birth, severe infections, and birth asphyxia [2]. Most of these deaths occur in developing countries. There is dearth of reliable information on causes of these deaths through routine vital registration systems. Furthermore, most deaths in these countries occur at home, thus hospital based medical certification of death is not available. This has important bearing on resource allocation and strategic planning [2]; [3].

Verbal Autopsy (VA) technique has been used in such situations to ascertain causes of child deaths [4] and neonatal deaths [5]. However, lack of standardised VA instrument and administration methods are the key challenges that remain unresolved [6]. Diagnostic accuracy of VA depends upon the VA tool, its administration, coding and classification of deaths, and cause specific mortality fractions in a particular area. Many VA studies have been done using different tools and classification systems. Validation studies with standard WHO VA tools have shown reasonable sensitivity and specificity for childhood deaths [4], [7]–[9], however diagnostic accuracy for neonatal deaths was poor [4], [10]–[12]. Therefore, a new verbal autopsy tool for neonatal deaths was developed by WHO [13]. This study was conducted to evaluate the performance of the WHO verbal autopsy tool in identifying the major causes of neonatal deaths in comparison with those assigned by paediatricians using standardised clinical and supportive radiology and laboratory data collected prospectively in the hospitals.

The study was approved by the Postgraduate Institute of Medical Education and Research Ethics Committee vide approval letter number EC -05/330 dated 31.10.2005 and WHO’s Ethics Review Committee vide protocol ID CHD 05010, meeting date 2005-11-10. Respondent’s information was kept confidential.

## Materials and Methods

The neonatal deaths were prospectively enrolled during 2006–2008 from a tertiary care neonatal unit and a government multi-speciality hospital of Chandigarh, a city in northern India. The study sites were selected in a WHO proposal development workshop where research teams of Bangladesh, Ghana, India and Pakistan had participated and selection was based on the quality of the proposals, neonatal mortality rates and the experience of the research team in evaluating verbal autopsy in the four candidate countries. At the time of this study, infant mortality rate was 55/1000 live births in Haryana, 44/1000 live births in Punjab and 23/1000 live births in Chandigarh [14]. Clinical diagnosis assigned by paediatricians of the study hospitals were considered gold standard diagnosis as they were trained to use guidelines for assigning cause of death using clinical, laboratory, radiological or any other investigations. Study hospitals are the leading hospitals in the region with good clinical care, record keeping and laboratory facilities. The distribution of causes of neonatal deaths was similar to the other published data from the country.

### Study Period

Hospital data collection was done from 15^th^ April 2006–31^st^ March 2008. Field data was collected from 1st June 2006 to 30th April 2008.

### Inclusion Criteria

All neonatal deaths that occurred in the study hospitals from 15^th^ April 2006–31^st^ March 2008, for which clinical information was obtained within 2 days of death were included in the study. This criterion was kept to minimise recall bias for making the gold standard diagnosis based on the information from clinical examination, laboratory, radiological and other investigations.

### Enrolment of Neonatal Deaths

**Figure 1:**
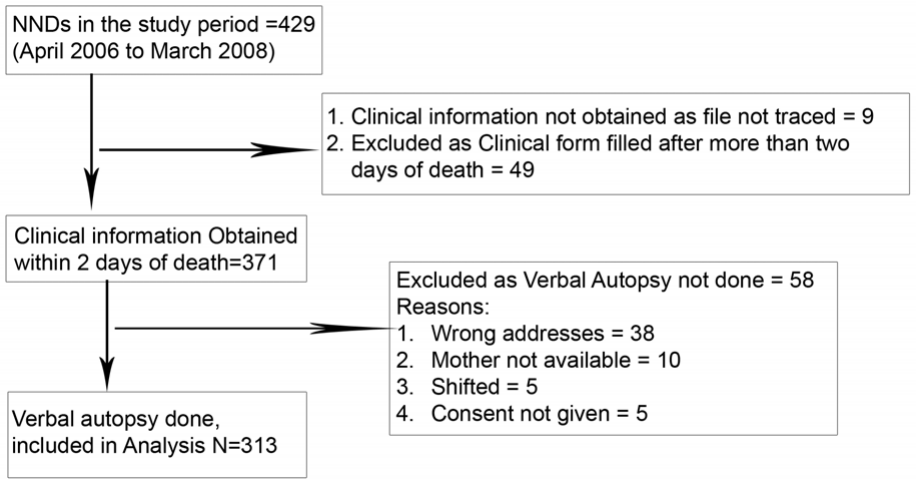
Book Keeping for Neonatal Deaths.

A total of 429 neonatal deaths occurred during the study period. Clinical information was obtained within 2 days of death in 371 cases and verbal autopsy could be performed in 313 cases. Verbal autopsy could not be done in 58 cases as either family was not traceable or families refused to give consent (figure 1).

### Study Tools

A structured clinical case sheet was used to record the maternal history for any underlying chronic conditions, obstetric history, antenatal history, examination and investigation findings, details of labour and delivery, findings of newborn’s examination at birth, details of history and examination of the neonate at admission, detailed structured examination findings, and investigation results. Follow up notes were recorded on a follow up sheet. Clinical investigators used this form to fill hospital based death certificate and used case definitions given in the appendix S1.

To conduct verbal autopsy, we used WHO verbal autopsy tool for neonatal deaths that was slightly modified to make it culturally more sensitive and deleting questions pertaining to health care seeking behaviour. Questions relating to health care seeking behaviour were deleted as the objective of the project was to ascertain accuracy of the VA tools only for the medical causes of death. Questions were made culturally appropriate to include locally relevant terms. For example to get the details of birth asphyxia, diarrhoea, pneumonia, tetanus etc. different terms were used in local vernacular language. These local terms were captured with discussion amongst the staff members having extensive field experience in the local area. Original questionnaires were in English language. English version was edited to include the local terms. After that two different persons translated the English questionnaire into Hindi and Punjabi language that are the common languages of the region. These questionnaires were translated back into English language by different set of persons. This retranslated version was compared with the original English version. Item-wise comparison was done to note if any item of the retranslated version is leading to a different meaning. Translated versions were then pretested before final use in the study.

Verbal autopsy (VA) tools had open-ended narrative part to note verbatim account of respondent’s version regarding the illness preceding the death; and a structured part to ask disease specific questions from the respondent. Main questionnaire had 10 sections to record interview’s details, respondent characteristics, age and place of death, narrative verbatim account of the illness preceding the death, maternal history during pregnancy labour and delivery, complications that occurred during labour and delivery, newborn’s details at birth and its status after birth, events immediately after birth like -was baby able to breathe immediately after birth, any assistance given to the baby to help him/her breath, questions on cry at birth, details of neonatal illness that led to death, and any treatment received. VA based death certificate was filled after reviewing the completed VA questionnaire. Definitions for assigning causes of deaths based on verbal autopsy are given in appendix S2.

### Death Certificates

Both clinical and verbal autopsy death certificates were divided into two parts. Part 1 was used to record the direct cause of death and up to three antecedent causes. Part II had two subparts: i) to record any other significant condition that might have contributed to the NND but not related to the disease or condition causing it and ii) to record any maternal condition that might have contributed to the neonatal death. In the end, there was space to write single underlying cause of neonatal death and the underlying maternal condition, if any.

### Training of the Study Team

Project staff was trained for data collection, data validation, data entry, and supervision. Field workers and field supervisors were shown videos of neonatal illnesses as used in Integrated Management of Neonatal and Childhood Illnesses (IMNCI) training programmes to make them aware about the neonatal health issues, and subsequently each and every question in the VA questionnaire was discussed. A field guide, explaining how to ask each question, was used in the training programme. Subsequently, each worker did at least two role plays. When field workers were comfortable in taking informed consent and asking each question, then they did two VAs in the field, for the deaths in the presence of one of the investigator (AKA). It took two weeks to complete this training. Supervisors were given similar training for conducting VA. In addition they were trained to check the forms. They were given the forms to detect the discrepancies that were pre-introduced by the investigator. Data manager was given training to design data entry forms in EPI info computer package and use validation checks. Data manager was trained to generate weekly analysis reports with frequency tables and cross tabulations to detect any data entry error. He was also trained to cross check the entire data with 10% of the original forms.

Project medical officers were trained to extract the information from the clinical files. Clinical investigators were imparted training to assign clinical causes of deaths. Training of clinical investigators was directly done by a WHO official. In periodic review meetings, the causes of deaths assigned by them were reviewed in his presence, and they were reoriented to have common understanding about definitions to be used for assigning the causes of neonatal deaths.

### Enrolment of Neonatal Deaths in the Hospital

Two trained project doctors visited Neonatal Intensive Care Unit (NICU) of tertiary care institute and paediatrics emergency of govt. multi-speciality hospital of Chandigarh on all working days and enrolled total of 429 neonatal deaths and could get clinical information within 2 days of occurrence of death in 371 cases. They discussed these cases with the treating doctors to fill the gaps in the clinical history and laboratory details and transferred the complete information on the hospital summary form. Clinical co-investigators used this information and followed standard clinical case definitions (Appendix S1) to make the clinical diagnosis. They used hierarchical classification of cause of deaths (Appendix S3) and ICD 10 classification, to assign the causes of death on death certificate.

### Verbal Autopsy

One month after the death, trained field workers with 10 years of school education visited 371 families to do the verbal autopsy. In case family was not available on first visit, one repeat visit was done. However, in 58 cases VA could not be done due to reasons mentioned in figure 1. Thus VA information obtained in 313 cases was included in the final analysis. Informed written consent of the respondents was taken before the conduct of the interview. Verbal autopsy interviews were done in the local language of the respondents (Hindi or Punjabi).

Two independent paediatricians, who were not involved in providing clinical care to the deceased, had no access to the clinical case files and were blinded to the cause of deaths assigned by the clinical team, independently reviewed the Verbal Autopsy forms. They assigned causes of death as per the standard definitions (Appendix S2) using ICD 10 classification, and completed the death certificates. The underlying causes assigned by the two paediatricians were compared. In cases of disagreement between the two, a third paediatrician assigned the cause of death. Agreement of two out of the three paediatricians was considered as final underlying cause.

### Quality Assurance

Weekly review meetings and supervisory field visits were done. Investigator drew random sample of 10% deaths every week, using random number table. Supervisors visited the sampled houses to check whether these houses were actually visited by the field workers and interview was done with appropriate respondents (mothers in most cases, or other adult respondent in case mother was not available and who was available at the time of death). Field workers pre-informed the families at the time of their visit that their supervisor might visit them. Supervisors took telephonic pre-consent from the families, wherever phone numbers were available. After reaching the family, verbal consent was taken again before the re-interview. First, it was confirmed that whether someone had visited them and filled a form for the deceased neonate. Then they were assured that the information was being again collected to check how correct the information collected by the field worker was. Key questions from the supervisory forms were compared with the field workers’ forms. Discrepancies if any in the repeat interviews were discussed in every weekly meeting with the entire team for continuous quality improvement in data collection and recording.

Field monitoring visits were conducted by the WHO official (World Health Organisation, Department of Child and Adolescent Health Division) to observe the quality of data collection processes at the hospital and the field level.

### Confidentiality and Data Security

Identification details of the deceased and the respondents were kept confidential. All forms were kept under lock and key. Data back-up was kept at three places in three different buildings.

### Statistical Analysis

Cause specific mortality fractions were calculated for each underlying cause of neonatal death using clinical diagnosis as well as verbal autopsy diagnosis. Sensitivity, specificity, positive predictive value and negative predictive value estimate with 95% confidence interval were calculated for the four most common underlying causes of neonatal deaths as per verbal autopsy, using the clinical diagnosis as the gold standard. Diagnostic accuracy was also calculated for each of these four causes of deaths separately and also for all the four causes together.

Calculations were repeated after re-categorization of causes of death allowing for multiple causes of each death. In this re-categorization, if any of these causes of neonatal death was recorded in the verbal autopsy death certificate as a direct, antecedent or underlying cause of death by any of the two reviewers; it was considered to be a cause of death and was compared with the clinical diagnosis. Area under Receiver Operating Characteristics (ROC) curve was calculated with both types of categorization. ROC shows the trade off between sensitivity and specificity. Adequate performance of verbal autopsy tool was considered to have area under ROC of at least 0.75, and a sensitivity above 60% and specificity above 85% [15]–[16]. Further, Kappa statistics was calculated to ascertain agreement between hospital and verbal autopsy diagnosis.

Cause specific mortality fractions of 58 neonatal deaths, as per hospital diagnosis, for which verbal autopsy could not be conducted were compared with the CSMF of 313 deaths to note if they had different distribution of causes of deaths. Further, key parameters namely neonatal age, maternal gestation period, mean birth weight, and multiple births of neonates in whom VA was done, was compared with that of 58 neonatal deaths in whom VA could not be done, to ascertain if there was any systematic difference between these two groups. This information was taken from the hospital records.

## Results

A total of 429 neonatal deaths occurred during the study period. Of these 371 deaths met the inclusion criteria in which clinical information was obtained within 2 days of death. Of these VA was performed in 313 cases, as explained above. Final analysis is based on these 313 cases in whom VA was performed. Key parameters of these children in whom VA was done were compared with those where VA could not be done to rule out any systematic difference between the two groups. The two groups were not statistically different with respect to the neonatal age, maternal gestation period, mean birth weight, and multiple births.

### Distinguishing Neonatal Deaths and Stillbirths

One neonatal death was considered as stillbirth during verbal autopsy. As per clinical assessment baby had congenital anomaly, and baby did not cry after birth, was born at 32 weeks gestation with birth weight of 1540 grams and had APGAR score of less than 4 at 5 minutes. Baby died within first hour of birth.

### Cause Specific Mortality Fractions

Preterm birth, sepsis, asphyxia and congenital anomalies were four leading causes that covered 88% of the neonatal deaths (Table 1). The proportion of neonatal deaths due to birth asphyxia was higher with clinical assessment compared to VA. In about 9% of the cases cause of death remained unexplained on VA. Clinical based cause specific mortality fractions for 313 NNDs that were included in the study were not statistically different from that of 58 NNDs deaths that could not be included as VA could not be done in these cases.

**Table 1:** Cause Specific Mortality Fractions for Neonatal Deaths as per Clinical and Verbal Autopsy Diagnosis.

SNO	Diagnosis Category	Clinical Diagnosis (N = 313)		Verbal Autopsy Diagnosis (N = 313)		p-value
		N	%	N	%	
1	Congenital Anomaly	36	11.5	25	8.0	0.18
2	Preterm birth	87	27.8	94	30.0	0.79
3	Asphyxia	85	27.2	51	16.3	<0.01
4	Sepsis	91	29.1	105	33.5	0.14
5	Other Specific Conditions	13	4.1	10	3.2	0.17
6	Unexplained	1	0.32	28	8.9	0.00

As per clinical assessment, 23.1% of neonatal deaths due to infection and 27.1% of neonatal deaths due to perinatal asphyxia were preterm.

### Diagnostic Accuracy of VA

Diagnostic accuracy of verbal autopsy diagnosis was estimated for the leading four causes, each of which contributed to over 10% of neonatal mortality by clinical diagnosis. The diagnostic accuracy of VA ranged from 78% to 92% for the different causes. Area under ROC for four major causes of NNDs, i.e., sepsis, preterm birth, congenital anomaly, and perinatal asphyxia was 0.70–0.75. However, VA could only correctly identify about half the deaths due to congenital anomalies and asphyxia (Table 2). Kappa agreement for all four causes was moderate (0.46–0.55). Kappa agreement dropped off from moderate to fair for infections, when scope of VA diagnosis was expanded from single underlying cause to multiple causes that is, underlying cause, direct cause, antecedent cause or contributory cause made by any of the two reviewers was included. Similarly, sensitivity improved and specificity dropped for all the four causes with the expansion of the scope of diagnosis to include multiple causes (Table 3).

**Table 2:** Validity of verbal autopsy based assignment of a single underlying cause * of neonatal deaths compared with the cause of death assigned by the treating neonatologist based on clinical and laboratory information.

Diagnosis Category	Sensitivity		Specificity		Positive Predictive Value	Negative Predictive Value	Diagnostic Accuracy	Area under ROC	Kappa Agreement
	N	%	N	%	%	%	%	Proportion	Agreement
		(95% CI)		(95% CI)	(95% CI)	(95% CI)	(95% CI)	(95% CI)	(95% CI)
Congenital Anomaly	36	50	277	97.5	72.0	93.8	92.0	0.73	0.55
		(33.2–66.8)		(94.6–98.9)	(50.4–87.1)	(90.1–96.1)	(88.4–94.7)	(0.65–0.82)	(0.39–0.70)
Preterm birth	87	65.5	226	83.6	60.6	86.3	78.6	0.74	0.48
		(54.5–75.2)		(78.0–88.1)	(50.0–70.4)	(80.9–90.4)	(73.6–83.0)	(0.69–0.80)	(0.37–0.59)
Asphyxia	85	45.9	228	94.7	76.5	82.4	81.5	0.70	0.46
		(35.1–57.0)		(90.8–97.1)	(62.2–86.8)	(77.2–86.7)	(76.7–85.6)	(0.65–0.76)	(0.35–0.58)
Severe infection(pneumonia, sepsis or meningitis)	91	69.2	222	81.1	60.0	86.5	77.6	0.75	0.48
		(58.6–78.3)		(752–85.9)	(50.0–69.3)	(81.0–90.7)	(72.6–82.1)	(0.70–0.80)	(0.38–0.58)

* Physicians coding verbal autopsy training in assigning underlying cause of death based on ICD10 classification.

**Table 3:** Validity of verbal autopsy based assignment of any cause* of neonatal deaths compared with the cause of death assigned by the treating neonatologist based on clinical and laboratory information.

Diagnosis Category	Sensitivity		Specificity		Positive Predictive Value	Negative Predictive Value	Diagnostic Accuracy	Area under ROC	Kappa Agreement
	N	%	N	%	%	%	%	Proportion	Agreement
		(95% CI)		(95% CI)	(95% CI)	(95% CI)	(95% CI)	(95% CI)	(95% CI)
Congenital Anomaly	36	61.1	277	95.7	68.8	95.0	92.3	0.78	0.58
		(43.5–76.4)		(92.4–97.6)	(49.9–83.3)	(91.6–97.1)	(88.8–95.0)	(0.71–0.87)	(0.44–0.73)
Preterm birth	87	81.6	226	69.0	50.4	90.7	72.5	0.75	0.43
		(71.6–88.8)		(62.5–74.9)	(41.9–58.8)	(85.1–94.4)	(67.2–77.4)	(0.70–0.80)	(0.33–0.57)
Asphyxia	85	54.1	228	89.0	64.8	83.9	79.6	0.72	0.45
		(43.0–64.9)		(84.1–92.6)	(52.5–75.5)	(78.5–88.2)	(74.6–83.9)	(0.66–0.77)	(0.34–0.57)
Severe infection (pneumonia, sepsis or meningitis)	91	78.0	222	66.2	48.6	88.0	69.6	0.72	0.37
		(67.9–85.8)		(59.5–72.3)	(40.3–57.0)	(81.9–92.4)	(64.2–74-7)	(0.67–0.77)	(0.28–0.47)

As per clinical diagnosis, in 50.6% of deaths due to preterm births, 47.1% due to perinatal asphyxia and, there was an identifiable maternal condition that could have resulted in the neonatal death (Table 4). In 39.6% neonatal deaths due to severe infections, there were some associated maternal conditions like Pregnancy Induced Hypertension (PIH), multiple pregnancy, antepartum haemorrhage etc. In 8 cases there was underlying maternal infection. By verbal autopsy, respective figures for preterm birth, perinatal asphyxia and severe infections were 69.1%, 51.0% and 56.2% (Table 5).

**Table 4:** Maternal condition that could have resulted in the underlying cause of neonatal death based on clinical and laboratory information.

Underlying neonatal cause of death (Clinical)	Maternal condition that could have resulted in the neonatal cause of death (Clinical)							
	Multiple pregnancy n(%)	Maternal disease existing before pregnancy n(%)	Pregnancy induced hypertension n(%)	Antepartum haemorrhage n(%)	Obstetric complications n(%)	Maternal Infections/Chorioamnioits n(%)	Other Specific conditions n(%)	No maternal conditionn(%)
Preterm birth (n = 87)	7 (8.0)	5 (5.7)	11(12.6)	12(13.8)	5 (5.7)	2 (2.3)	2 (2.3)	43 (49.4)
Asphyxia (n = 85)	1(1.2)	4(4.7)	14(16.5)	10(11.8)	5(5.9)	5(5.9)	1(1.2)	45(52.9)

**Table 5:** Maternal condition that could have resulted in the underlying cause of neonatal death based on verbal autopsy.

Underlying neonatal cause of death (VA)	Maternal condition that could have resulted in the neonatal cause of death (VA)						
	Multiple pregnancy n(%)	Maternal disease existing before pregnancy n(%)	Pregnancy induced hypertension n(%)	Antepartum haemorrhagen(%)	Obstetric complications n(%)	Other specific conditions n(%)	No maternal condition n(%)
Preterm birth (n = 94)	21(22.3)	4(4.3)	13(13.8)	22(23.4)	0	5(5.3)	29(30.9)
Asphyxia (N = 51)	0	3(5.9)	6(11.8)	12(23.5)	2(3.9)	3(5.9)	25(49.0)

## Discussion

Main findings of our study are that congenital malformations, preterm births, perinatal asphyxia and severe infections accounted for about 90% of all neonatal deaths. CSMF as per clinical diagnosis are similar to those as per VA for all major causes of neonatal death except perinatal asphyxia. Our findings indicate that VA substantially underestimates deaths due to perinatal asphyxia.

VA had acceptable level of diagnostic accuracy for all four major causes of neonatal deaths. The cause-specific mortality fraction has an important influence on the size of the error for given levels of sensitivity and specificity, and when the cause-specific mortality fraction is small, size of the error depends more on the specificity than sensitivity [17].

The low sensitivity of VA in diagnosing a death due to congenital anomaly and perinatal asphyxia means that up to half the deaths due to these conditions are not correctly identified by VA. Possible reasons could be that, VA is not expected to accurately diagnose several major congenital anomalies, e.g. congenital heart defects. It will probably be able to diagnose well only *visible* anomalies such as anencephaly and spina bifida.VA is also not expected to be great in identifying perinatal asphyxia because the mothers are unlikely to be aware of all the newborn events immediately after birth, particularly in hospital settings where the baby is taken away for care after delivery.

Currently, most studies based on verbal autopsy assign a single underlying cause of death [18]. However, some experts have suggested that this may not be the most appropriate strategy and multiple causes of deaths should be considered [4]. When we considered multiple causes, the sensitivity of verbal autopsy increased for preterm births, congenital anomaly, birth asphyxia and sepsis; however, there was substantial reduction in specificity for preterm births and sepsis (table 2 and 3). Marsh etal (2003) also reported increase in sensitivity for birth asphyxia with multiple causes of death [12]. Lee et al (2008) also had similar observations [19]. In our study sensitivity for preterm births and sepsis was better and for birth asphyxia it was comparable to other studies except the study by Edmond etal (2008) who found that sensitivity of VA was >60% for all major causes of neonatal deaths and specificity was 76% for birth asphyxia but >85% for prematurity and infection [18]. Edmond et al were probably working at district level hospitals without many lab facilities etc. Therefore VA and hospital diagnosis may have been similar. This may also be because of our greater sample size and choice of two different levels of hospitals catering to different types of case loads. Higher sensitivity for sepsis in our study is unlikely to be because of different case definition, as for making clinical diagnosis of sepsis we also required at least two of the following clinical signs of sepsis to be present in the neonate: (fever or hypothermia, convulsions, not feeding well, no spontaneous movement, weak or absent cry, abdominal distension). Birth asphyxia although had low sensitivity but area under ROC was fair, and Kappa agreement was moderate.

In this study paediatricians assigned the causes of death both for clinical diagnosis and for verbal autopsy using standard definitions and guidelines. Chances of classification bias in these two teams were negligible because uniform training protocols were followed. Moreover, clinical investigators were involved in the clinical care, and they consulted the primary clinical record before assigning the cause of death, that was taken as gold standard. For verbal autopsy diagnosis, there are two recommended methods for review and consensus building among different reviewers i) discussion and consensus building among reviewers and ii) another independent review by third reviewer and agreement of any two out of three. We followed the second approach, as in the first, there are chances of one reviewer getting influenced by other as a mark of respect for seniority or otherwise. Second approach gives more weight for the independent decisions of the three physicians and we therefore chose it over the first one.

Some differences in cause specific mortality fractions of clinical diagnosis with verbal autopsy are noteworthy. Clinical diagnosis assigned a greater proportion of neonatal deaths due to asphyxia. We believe it may be because birth asphyxia requires respondents to recall the events at the time of birth in the labour rooms. It might be difficult for the mothers to recall such events as they themselves are in distress and the relatives are usually not allowed in the delivery rooms. Situation is more difficult if some prior medications are given before delivery or caesarean sections or if the respondent was anybody else than the mother in case of maternal mortality. On the other hand detailed information is available with the clinicians to make clinical diagnosis. Despite this, it is remarkable that the cause specific mortality fractions for most causes of neonatal deaths were so similar with clinical and verbal autopsy diagnosis. Moreover, million death study in India, that used verbal autopsy method in community setting also arrived at similar estimates [20]. Diagnosis remained unexplained in 28 (8.9%) of the cases on VA. Of these 28 cases, 6 had congenital anomaly, 4 were preterm, 8 had birth asphyxia and another 3 had died due to other specific conditions diagnosed as per hospital diagnosis. VA history did not provide sufficient information for these events that were largely around the time of birth, for making a probable diagnosis. Lack of information was due to inability of respondents to provide sufficient details, not due to suboptimal quality of the data collection, as quality assurance protocols for data collection were very stringent.

VA overestimated multiple pregnancy and antepartum hemorrhage as contributory maternal conditions for preterm births, and pregnancy induced hypertension and antepartum hemorrhage for severe infections. It underestimated pregnancy induced hypertension as a contributory maternal condition for perinatal asphyxia.

There are several strengths of this study. This was a large, prospective, well designed validation study. There were efforts to make the cause of death assignment from clinical and lab information as close to “gold standard” as possible, including (i) treating neonatologists were trained in completing death certificates based on ICD principles (ii) a research officer ensured that the clinical and lab information was reviewed and a final death certificate was completed by a treating consultant neonatologist within 2 days of death.

Assignment of verbal autopsy diagnosis was well standardized (i) a standard WHO questionnaire was used to conduct VA (ii) training and standardization of the VA team in conducting the interview (iii) pediatricians who assigned causes of death after reading the VA questionnaire were trained in completing death certificates based on ICD principles (iv) the standard death certificate was completed for each VA.

This is first VA study to assess and report maternal conditions contributing to three major causes of neonatal deaths: immaturity, perinatal asphyxia and severe infections.

Some limitations of the study also merit consideration. First, the study enrolled neonatal deaths from the hospital setting. It may be argued that verbal autopsy validation results based on a hospital based study might not be applicable to that in the general population because of the differences in the cause structure of the validation sample with that of the general population, and also because of the differences in the quality of recall in the two population groups. Provider interactions may influence recall. However, it is noteworthy that it is not possible to conduct a validation study in the community because of lack of an acceptable “gold standard” cause of death. Secondly, we used only one method of assigning cause of death by VA, namely a review by a panel of paediatricians. Several methods have been reported in the literature, including physician review, pre-defined computer algorithms and probabilistic models [16], [21]–[23]. However, the most commonly used method for interpreting VA remains review by a panel of physicians. Computer based algorithms hold promise in future. However, at the conduct of this study, most of the experience with computer based algorithms was restricted to adult VAs [22]. Even in these studies computer based algorithms was recommended as alternate if physician review is not possible.

The experience of using computer algorithm for neonatal VAs was found to be equivocal at the time of conduct of this study. Freeman *et al* found the results of physician review and computer based algorithms were disparate for some causes like congenital anomaly, prematurity and birth asphyxia, that are the leading causes of deaths in the study population [24]. The largest experience of using VA as of today is in million deaths study. Even this required involvement of physicians to review the deaths. Computer algorithms could be an aid to the physicians in the review to reduce the time taken for assigning the causes.

Conducting interviews 4–6 weeks after the neonatal death may introduce bias. However from the ethical perspective mourning period of at least one month should be allowed before the interviews. Conducting interviews very late may influence recall however, at 4–6 weeks recall based on communication with hospital staff is likely to lessen, whereas they may still be able to recall the symptoms and signs for the illness preceding death. In this study a panel of paediatricians assigned the causes of death by VA using standard definitions as guidelines and a hierarchical classification. There are arguments that favour using general physicians for this purpose, as specialists may have their own preferences with respect to the clinical diagnosis [25]. However, we chose paediatricians for our validation study to ensure that the training and experience of those who assigned causes of death in the hospital and by verbal autopsy should be similar to prevent reviewer’s bias in classification of causes of deaths. We used the commonly used method of using a third reviewer to settle any disagreement among the two reviewers by agreement of two out of three. We did not attempt to achieve consensus by discussion to avoid the reviewer with seniority within the organization unduly influencing the decision. Further, most differences between reviewers were related to what they chose to be the single underlying cause of death. On considering multiple causes of deaths, the differences were minimal.

Our study has also given insights into maternal conditions that were associated with and probably contributed to preterm birth and birth asphyxia. For NNDs due to severe infections/sepsis, there were maternal conditions that were found to be associated. In most of these deaths, babies were either preterm or low birth weight. In few deaths there were underlying maternal infections/chorioamnioitis.

Lastly, VA could not be done for about 15% deaths - but CSMF in this group was similar to those included in the analysis. Furthermore, key characteristics of these neonates were similar to those who were included in the study.

In conclusion, verbal autopsy tools provide reasonably good estimates about predominant causes of NNDs like Sepsis, Prematurity, Congenital Anomaly, and Birth Asphyxia in the setting where cause specific mortality fraction is high due to these conditions. Use of multiple causes of death gives relatively better diagnostic accuracy of verbal autopsy compared to the use of single underlying cause. Further validation studies in other populations and geographic areas will help in generalisation of the findings related to validity of verbal autopsy tools for ascertaining the causes of NNDs.

## Supporting Information

Appendix S1 **Definitions for causes of Neonatal Death Certification from Hospital data.**
(DOCX)

Appendix S2 **Definitions for causes of Death Certification from Verbal Autopsy.**
(DOCX)

Appendix S3 **List of Causes of Neonatal Deaths.**
(DOCX)

## References

[1] World Health Organisation (2005) World Health Report 2005. Make every mother and child count. Geneval Switzerland: WHO.

[2] Lawn JE, Wilczynska-Ketende K, Cousens SN (2006) Estimating the causes of 4 million neonatal deaths in the year 2000. Int J Epidemiol Jun 35(3): 706–18. 10.1093/ije/dyl043 16556647

[3] Lawn JE, Osrin D, Adler A, Cousens S (2008) Four million neonatal deaths: counting and attribution of cause of death. Paediatr Perinat Epidemiol Sep 22(5): 410–6. 10.1111/j.1365-3016.2008.00960.x PMC3428888 18782248

[4] Anker M, Black RE, Coldham C, Kalter HD, Quigley MA, et al.. (1999) A Standard Verbal Autopsy Method for Investigating Causes of Death in Infants and Children. Geneva World Health Organisation; Report No.: WHO/CDS/CSR/ISR/99.4.

[5] Baqui AH, Darmstadt GL, Williams EK, Kumar V, Kiran TU, et al (2006) Rates, timing and causes of neonatal deaths in rural India: implications for neonatal health programmes. Bull World Health Organ Sep 84(9): 706–13. 10.2471/blt.05.026443 PMC2627477 17128340

[6] Thatte N, Kalter HD, Baqui AH, Williams EM, Darmstadt GL (2009) Ascertaining causes of neonatal deaths using verbal autopsy: current methods and challenges. J Perinatol Mar 29(3): 187–94. 10.1038/jp.2008.138 19110535

[7] Kalter HD, Gray RH, Black RE, Gultiano SA (1990) Validation of postmortem interviews to ascertain selected causes of death in children. Int J Epidemiol Jun 19(2): 380–6. 10.1093/ije/19.2.380 2376451

[8] Quigley MA, Armstrong SchellenbergJR, Snow RW (1996) Algorithms for verbal autopsies: a validation study in Kenyan children. Bull World Health Organ 74(2): 147–54. 8706229 PMC2486900

[9] Snow RW, Armstrong JR, Forster D, Winstanley MT, Marsh VM, et al (1992) Childhood deaths in Africa: uses and limitations of verbal autopsies. Lancet Aug 8 340(8815): 351–5. 10.1016/0140-6736(92)91414-4 1353814

[10] Coldham C, Ross D, Quigley M, Segura Z, Chandramohan D (2000) Prospective validation of a standardized questionnaire for estimating childhood mortality and morbidity due to pneumonia and diarrhoea. Trop Med Int Health Feb 5(2): 134–44. 10.1046/j.1365-3156.2000.00505.x 10747274

[11] Kalter HD, Hossain M, Burnham G, Khan NZ, Saha SK, et al (1999) Validation of caregiver interviews to diagnose common causes of severe neonatal illness. Paediatr Perinat Epidemiol Jan 13(1): 99–113. 10.1046/j.1365-3016.1999.00151.x 9987789

[12] Marsh DR, Sadruddin S, Fikree FF, Krishnan C, Darmstadt GL (2003) Validation of verbal autopsy to determine the cause of 137 neonatal deaths in Karachi, Pakistan. Paediatr Perinat Epidemiol Apr 17(2): 132–42. 10.1046/j.1365-3016.2003.00475.x 12675779

[13] World Health Organisation (2007) Verbal autopsy standards : ascertaining and attributing cause of death. 2012. 20 Avenue Appia, 1211 Geneva 27, Switzerland, WHO Press, World Health Organization. 8-5-2012.

[14] Registrar General of India (2007) SRS Bulletin, October 2007. Census and Vital Statistics Website on the Internet. 42 [1]. 2012. New Delhi, Office of the Registrar General, India, Ministry of Home Affairs, Govt. of India, 2-A Mansingh Road, New Delhi 110 011, India. 8-5-2012.

[15] World Health Organisation (2005) International statistical classification of diseases and related health problems. 2nd[10th revision]. Geneva, WHO.

[16] Winbo IG, Serenius FH, Dahlquist GG, Kallen BA (1998) NICE, a new cause of death classification for stillbirths and neonatal deaths. Neonatal and Intrauterine Death Classification according to Etiology. Int J Epidemiol Jun 27(3): 499–504. 10.1093/ije/27.3.499 9698143

[17] Anker M (1997) The effect of misclassification error on reported cause-specific mortality fractions from verbal autopsy. Int J Epidemiol Oct 26(5): 1090–6. 10.1093/ije/26.5.1090 9363532

[18] Edmond KM, Quigley MA, Zandoh C, Danso S, Hurt C, et al (2008) Diagnostic accuracy of verbal autopsies in ascertaining the causes of stillbirths and neonatal deaths in rural Ghana. Paediatr Perinat Epidemiol Sep 22(5): 417–29. 10.1111/j.1365-3016.2008.00962.x 18782250

[19] Lee AC, Mullany LC, Tielsch JM, Katz J, Khatry SK, et al (2008) Verbal autopsy methods to ascertain birth asphyxia deaths in a community-based setting in southern Nepal. Pediatrics May 121(5): e1372–e1380. 10.1542/peds.2007-2644 PMC2366089 18450880

[20] Bassani DG, Kumar R, Awasthi S, Morris SK, Paul VK, et al (2010) Causes of neonatal and child mortality in India: a nationally representative mortality survey. Lancet Nov 27 376(9755): 1853–60. 10.1016/S0140-6736(10)61461-4 PMC3042727 21075444

[21] Winbo IG, Serenius FH, Dahlquist GG, Kallen BA (1997) A computer-based method for cause of death classification in stillbirths and neonatal deaths. Int J Epidemiol Dec 26(6): 1298–306. 10.1093/ije/26.6.1298 9447410

[22] Quigley MA, Chandramohan D, Rodrigues LC (1999) Diagnostic accuracy of physician review, expert algorithms and data-derived algorithms in adult verbal autopsies. Int J Epidemiol Dec 28(6): 1081–7. 10.1093/ije/28.6.1081 10661651

[23] Fantahun M, Fottrell E, Berhane Y, Wall S, Hogberg U, et al (2006) Assessing a new approach to verbal autopsy interpretation in a rural Ethiopian community: the InterVA model. Bull World Health Organ Mar 84(3): 204–10. 10.2471/blt.05.028712 PMC2627286 16583079

[24] Freeman JV, Christian P, Khatry SK, Adhikari RK, LeClerq SC, et al (2005) Evaluation of neonatal verbal autopsy using physician review versus algorithm-based cause-of-death assignment in rural Nepal. Paediatr Perinat Epidemiol Jul 19(4): 323–31. 10.1111/j.1365-3016.2005.00652.x 15958155

[25] Setel PW, Whiting DR, Hemed Y, Chandramohan D, Wolfson LJ, et al (2006) Validity of verbal autopsy procedures for determining cause of death in Tanzania. Trop Med Int Health May 11(5): 681–96. 10.1111/j.1365-3156.2006.01603.x 16640621

