# Supplementary material for: Accuracy of WHO Verbal Autopsy Tool in Determining Major Causes of Neonatal Deaths in India
Source: PLoS One. 2013 Jan 25;8(1):e54865. doi: 10.1371/journal.pone.0054865 (PMC3555991; doi:10.1371/journal.pone.0054865)
Supplement: Appendix S1 — Definitions for causes of Neonatal Death Certification from Hospital data. (DOCX) [file pone.0054865.s001.docx]

**Appendix S1: Definitions for causes of Neonatal Death Certification from Hospital data**

Accidents/injuries: The cause of neonatal death can be clearly attributed to a severe accident or injury.

Congenital malformations: Only lethal or potentially lethal malformations that markedly increase mortality risk e.g. anencephaly, large meningomyelocoele, duodenal atresia, tracheo-oesophageal fistula or major cardiac malformations.

Prematurity: Gestation less than 33 weeks, and if gestation is not known or not reliable then birth weight <1.5 kg

*Hyaline membrane disease / Respiratory distress syndrome*: Presence of signs of respiratory distress (respiratory rate >60 per minute with either severe chest indrawing or grunting) in a preterm neonate with onset within 4 hours of birth. Chest radiograph shows poor lung expansion, reticulogranular pattern, ground glass opacity, and air bronchograms.

*Intra-venticular haemorrhage*: Radiological or ultrasonograhic evidence of intra-ventricular haemorrhage.

*Pulmonary haemorrhage*: Frothy fresh bleeding from nose or mouth.

Birth asphyxia: APGAR Score <4 at 5 minutes or <5 at 10 minutes alone is enough if death occurs within 6 hours of birth. For deaths beyond 6 hours of age, in addition to the low APGAR score as above, presence of at least one of the following neurological signs of Hypoxic Ischemic Encephalopathy (HIE) is mandatory: alteration in sensorium (hyper-alertness, jitteriness, lethargy or coma), seizures or hypotonia.

Neonatal tetanus: A physician’s clinical diagnosis of the signs of tetanus (lock jaw, back arching, convulsions, spasms) beginning on day 3 or later in a baby who was initially normal for the first 2 days of life.

Pneumonia: In a neonate with respiratory distress (respiratory rate >60 per minute with either severe chest indrawing or grunting), presence of at least one of the following:

- clinical signs of sepsis (see list below)

- positive sepsis screen

- chest X-Ray suggestive of pneumonia.

[Note: If onset is before 72 hours of life, presence of one of the following - maternal fever (during labour or within 3 days after birth), foul smelling liquor or prolonged rupture of membranes (>18 hours) is also necessary]

Meningitis: Positive lumbar puncture as defined as any of the following: culture or bacteria seen on gram stain, >32 leucocytes/mm^2^ in CSF with >80% PMNs

Sepsis: In a neonate with at least two of the following clinical signs of sepsis (fever or hypothermia, convulsions, not feeding well, no spontaneous movement, weak or absent cry, abdominal distension):

- Isolation of pathogen from blood: DEFINITE SEPSIS

- Absence of pathogen in blood culture but positive sepsis screen and clinical course suggestive of sepsis: LIKELY SEPSIS

- Absence of pathogen in blood culture and negative sepsis screen but clinical course suggestive of sepsis: POSSIBLE SEPSIS [Note: if onset is before 72 hours of life, additionally one of the following should be present - maternal fever, foul smelling liquor or prolonged rupture of membranes (>18 hours)]
