# Supplementary material for: Accuracy of WHO Verbal Autopsy Tool in Determining Major Causes of Neonatal Deaths in India
Source: PLoS One. 2013 Jan 25;8(1):e54865. doi: 10.1371/journal.pone.0054865 (PMC3555991; doi:10.1371/journal.pone.0054865)
Supplement: Appendix S2 — Definitions for causes of Death Certification from Verbal Autopsy. (DOCX) [file pone.0054865.s002.docx]

**Appendix S2: Definitions for causes of Death Certification from Verbal Autopsy**

Accidents/injuries: The cause of neonatal death can be clearly attributed to a severe accident or injury.

Congenital malformations: Only lethal or potentially lethal malformations that markedly increase mortality risk e.g. anencephaly, large meningomyelocoele.

Prematurity: Pregnancy lasted 7 months or less; or pregnancy lasted 8 months and the size of the baby was very small at birth.

Birth asphyxia: Baby did not cry or breathe immediately after birth, may have been given assistance to breathe. This history alone is enough if death occurs on the day of the birth. For deaths occurring after the day of the birth, presence of atleast one of the following starting on or before day 3 of life is also necessary with the above: convulsions, limp, or flaccid, unresponsive or unconscious.

Neonatal tetanus: Unable to open mouth after feeding well for the first days of life, has convulsions or spasms, beginning day 3 in a baby who was initially normal for the first 2 days of life.

Pneumonia: Fast breathing, chest indrawing or grunting, starting after day 3 of life.

Sepsis: At least two of the following clinical signs of sepsis- fever, cold to touch, stopped feeding well, lethargic or unresponsive/ unconscious after a period of normal activity, abdominal distension.
