# Supplementary material for: Accuracy of WHO Verbal Autopsy Tool in Determining Major Causes of Neonatal Deaths in India
Source: PLoS One. 2013 Jan 25;8(1):e54865. doi: 10.1371/journal.pone.0054865 (PMC3555991; doi:10.1371/journal.pone.0054865)
Supplement: Appendix S3 — List of Causes of Neonatal Deaths. (DOCX) [file pone.0054865.s003.docx]

**Appendix S3: List of Causes of Neonatal Deaths**

**Part: I ( Disease or Condition directly leading to death)**

1. Accident/ Injuries
2. Congenital Malformations
3. Prematurity (only if <33 weeks)
4. Birth Asphyxia
5. Tetanus
6. Pneumonia
7. Meningitis
8. Diarrhoea
9. Sepsis
10. Other Specific condition*
11. Unexplained neonatal death

*May not be possible to ascertain by verbal autopsy

**Part: II (Other Significant Conditions)**

**Infant Conditions**

1. Pre-term (33-36 weeks gestation)
2. Low birth weight
3. Others

**Maternal Conditions**

1. Multiple pregnancy
2. Maternal disease existing before pregnancy (Diabetes/ Hypertension/ Epilepsy/ Hepatosis/ Renal disease)
3. Pregnancy induced hypertension (pre-eclampsia/ Eclampsia)
4. Antepartum haemorrhage (Abruptio placentae/ Placenta praevia)
5. Obstetric complications (Malpresentation/ Cord prolapse/ obstructed labour/ uterine rupture)
6. Others
